# HLA‐DQβ1 amino acid position 87 and DQB1*0301 are associated with Chinese Han SLE

**DOI:** 10.1002/mgg3.403

**Published:** 2018-04-19

**Authors:** Jingying Sun, Chao Yang, Wenmin Fei, Xuelei Zhang, Yujun Sheng, Xiaodong Zheng, Huayang Tang, Wanling Yang, Sen Yang, Xing Fan, Xuejun Zhang

**Affiliations:** ^1^ Institute of Dermatology and Department of Dermatology at NO. 1 Hospital Anhui Medical University Hefei China; ^2^ Key Laboratory of Dermatology Anhui Medical University Ministry of Education Hefei China; ^3^ LKS Faculty of Medicine Department of Paediatrics and Adolescent Medicine The University of Hong Kong Pokfulam Hong Kong; ^4^ Department of Dermatology No. 2 Hospital Anhui Medical University Hefei China

**Keywords:** HAN, imputation, MHC, systemic lupus erythematosus

## Abstract

**Background:**

Several susceptibility loci have been identified associated with Chinese Han systemic lupus erythematosus (SLE).

**Methods:**

We carried out imputation of classical HLA alleles, amino acids and Single Nucleotide Polymorphisms (SNPs) across the MHC region in Chinese Han SLE genome‐wide association study (GWAS) of mainland and Hong Kong populations for the first time using newly constructed Han‐MHC reference panel followed by stepwise conditional analysis.

**Results:**

We mapped the most significant independent association to HLA‐DQβ1 at amino acid position (Phe87, *p *= 7.807 × 10^−17^) and an independent association at HLA‐DQB1*0301 (*P*_condiational_ = 1.43 × 10^−7^).

**Conclusion:**

Our study illustrates the value of population‐specific HLA reference panel for fine‐mapping causal variants in the MHC.

## 1 BACKGROUND

The major histocompatibility complex (MHC) region (chr 6:29–34 Mb) harbors the human leukocyte antigen (HLA) genes of which many are associated with autoimmune diseases (Fernando et al., 2008). The risk of autoimmunity conferred by HLA polymorphisms is likely the result of variation in amino acid residues at specific positions, which may alter the structure and function of presented peptides (Astill, Ellis, Arif, Tree, & Peakman, 2003; Lee, Wucherpfennig, & Wiley, 2001; van Lummel et al., 2014; Scally et al., 2013). For certain disease, specific amino acid positions within HLA molecules may play an important functional role. SLE is a heterogeneous disease characterized by autoantibody production and damage to multiple organs due to immune complexes and inflammation (Chung et al., 2011). A genetic contribution of the human leukocyte antigen (HLA) region to SLE has been supported by epidemiological studies and several genetic studies (Deng & Tsao, 2010; Lee et al., 2014). Genes in class II HLA regions are dominantly represented as SLE susceptibility loci especially in T cell dependent antibody responses. Professors have showen that HLA‐DRB1 was significantly associated with autoantibody subsets in SLE patients (Connolly & Hakonarson, 2012). Then studies fine‐mapped the primary association within the MHC locus with SLE to HLA‐DR, and further narrowed it down to specific amino‐acid positions. For example, the role of amino acid position 11‐13‐26 in HLA‐DRβ1 for Korean SLE susceptibility has been established (Kim et al., 2014). However, the effects of the genetic architecture of the MHC region on SLE risk have not yet to be fully elucidated due to the complexity of the region, extended regions of linkage disequilibrium (LD), and a lack of statistical power. Al‐Motwee et al. found that HLA‐DQB1*06 was associated with Saudis SLE patients (Al‐Motwee et al., 2013). SNP rs2187668 at HLA‐DR3 was significantly associated with antidsDNA and was stronger of association in anti‐dsDNA positive SLE subjects compared with negative ones (Chung et al., 2011). However, those loci don't fully explain the HLA mediated risk of SLE, and did not investigate the functional amino acids due to lack of a reference panel suitable for imputing their genetic variants. Data from GWAS also have had insufficient variant density to define the association signals within the MHC. In this study, we used recently established Han‐MHC reference panel (Zhou et al., 2016) imputing Chinese SLE GWAS data to identify potential independent amino acid positions.

## 2 METHODS

### 2.1 Ethical compliance

The study was approved by the relevant local Institutional Ethical Committees and informed consent was obtained from patients and families.

### 2.2 SLE GWAS data

We extracted genome‐wide SNPs of 1047 cases and 1205 controls subjects (Chinese mainland SLE, data set #1) and SNP data of 612 cases and 2193 controls individuals (Hong Kong SLE, data set #2) in previously SLE GWAS, and filtered using standard quality control criteria (Han et al., 2009; Yang et al., 2010) (Table S1), including SNP and sample call rate, exclusion of closely related relative and outliers in terms of ancestry, and SNP minor allele frequency (MAF) and Hardy–Weinberg equilibrium cutoffs.

### 2.3 HLA imputation

We imputed classical HLA alleles, HLA amino acid residues and untyped SNPs from each data set by SNP2HLA and the new Han‐MHC reference panel. Imputed markers with minor allele frequency (≥1%) and imputation quality (PLINK R2) ≥0.3 were used in disease association tests. Basing on the known amino acid sequences of classical alleles of HLA‐A, ‐B, ‐C, ‐DPA1, ‐DPB1, ‐DQA1, ‐DQB1 and ‐DRB1 in the IMGT/HLA database (database release 3.13.1) (Kallberg et al., 2007; Robinson et al., 2015) translated the amino acid residues of each HLA genes. All information about the SNPs, amino acid residues and two‐digit and four‐digit HLA alleles were encoded as binary variables and phased by Beagle 3.0.4 imputation program (Browning & Browning, 2009) powered by SNP2HLA (to extract SNP genotypes located in the MHC region) method (Jia et al., 2013).

All genotype data from the two data sets were merged as a single data set after excluding the SNPs that were not present in both data sets (*n* = 3,175 SNPs in mainland GWAS data; *n* = 2,885 SNPs in Hong Kong GWAS data; *n* = 6,060 SNPs in both panels). The study subject was highly homogenous in a principal component (PC) analysis (Gibbs et al., 2003).

### 2.4 Association analysis

For each phenotype, we assessed variant risk with a logistic‐regression model assuming additive effects of the allele dosages in the log‐odds scale. We defined HLA variants to include two‐ and four‐digit biallelic classical HLA alleles, biallelic HLA amino acid polymorphisms for respective residues, multiallelic HLA amino acid polymorphisms and biallelic SNPs across the entire MHC region. To account for potential population substructure, we included the top ten PCs and an indicator variable for each data set as covariates when examined SLE association of the imputed dosage of each marker with minor allele frequency of ≧1% and imputation quality (PLINK *R* ^2^) of ≧0.3 by logistic regression.

We also used a forward logistic regression model to find additional markers with independent SLE‐risk effect by adding the identified markers as covariates for conditional analysis.

### 2.5 Meta analysis

We used the inverse variance method for meta analysis, combining data from the two studies (Mainland and Hong Kong) for SNPs, alleles and amino acid with an imputation *R* ^2^ score of ≧0.3 in two studies.

## 3 RESULTS

### 3.1 HLA Imputation and association testing

After imputation of SLE GWAS data for Chinese Han subjects (1,659 cases versus 3,398 controls) we obtained genotypes for 10 HLA two‐digit alleles, 13 HLA four‐digit alleles, 173 HLA amino acid positions and 231 SNPs encoded by HLA genes of the class I and class II (*p* < 1.67 × 10^−6^) from the genotyped MHC SNPs (29–34 Mb at chromosome 6). We set a study‐wide significance threshold of *p* = 1.67 × 10^−6^ on the basis of the total number of imputed HLA variants (0.05/30,000).

### 3.2 Risk of HLA‐DQβ1 at amino acid position 87 associated with SLE subjects

**Figure 1:**
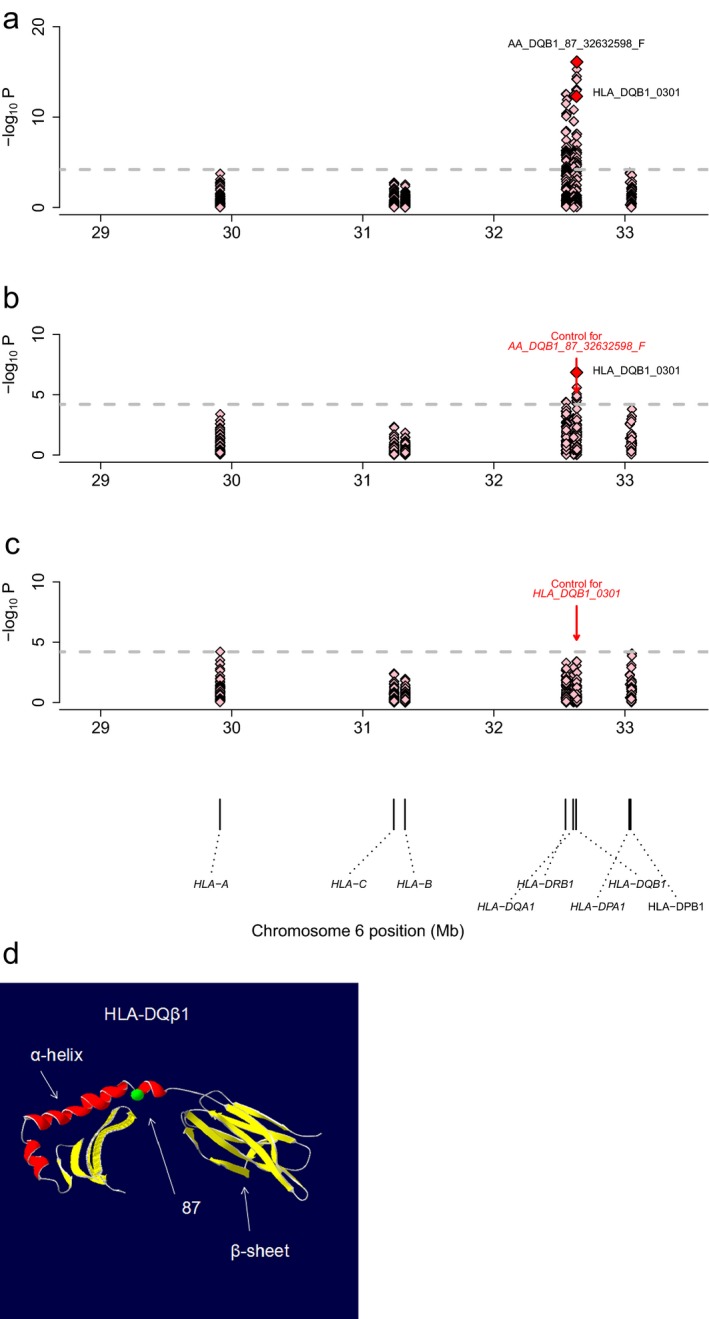
Association plots of the tested variants in the MHC region to SLE in Chinese Han Population. Each diamond represents ‐log_10_(P) of the variants, the classical HLA alleles and the amino acid polymorphisms of the HLA genes. The dotted horizontal line represents the significance threshold of *p* = 1.67 × 10^−6^. The bottom panel showed the physical positions of the HLA genes on chromosome 6 (NCBI Build 37). (a,b) Nominal associations in the SLE GWAS of Asians, in which HLA‐DQβ1 amino acid position 87 and HLA‐DQB1*0301 showed the first two most significant associations. (c) Conditional results on DQB1*0301, in which no variants showed significant associations. While amino acid polymorphisms reported in Hong Kong also showed better fitness of the model (position 87). (d) 3D ribbon models for the HLA‐DQB1. HLA‐DQB1 protein structure is based on SWISS‐MODEL entries 1uvq. The red one is a α‐helix, yellow one presents a β‐sheet and green spheres is HLA‐DQB1 amino acid position 87(F/Phe)

When testing the imputed HLA variants in the MHC region for association with SLE risk, we demonstrated the principal association with HLA‐DQB1. We assessed the risk associated with both classical HLA alleles and HLA amino acid polymorphisms, and by a meta‐analysis we found the most significant association was amino acid position 87 of HLA‐DQβ1 (*P*_meta_ = 7.81 × 10^−17^, OR = 1.785; Figure 1a; Tables 1, S1).

**Table 1:** Association results of the Top‐20 associated markers after HLA imputation

			Frequency [%]^d^				
Marker ID^a^	Position^b^	Alleles^c^	Patients	Control	P	OR^d^	Nearest gene
AA_DQB1_87_32632598_F	32632598	P/A	0.3031	0.1959	7.81E‐17	1.785	HLA‐DQB1
AA_DQB1_86_32632601_A	32632601	P/A	0.4345	0.3174	5.18E‐16	1.652	HLA‐DQB1
AA_DQB1_9_32632832_Y	32632832	P/A	0.3523	0.2448	3.05E‐15	1.678	HLA‐DQB1
AA_DQB1_140_32629890_A	32629890	P/A	0.3967	0.5129	6.30E‐15	0.6247	HLA‐DQB1
AA_DQB1_140_32629890_T	32629890	P/A	0.3967	0.5129	6.30E‐15	0.6247	HLA‐DQB1
AA_DQB1_182_32629764_N	32629764	P/A	0.3967	0.5129	6.30E‐15	0.6247	HLA‐DQB1
AA_DQB1_182_32629764_S	32629764	P/A	0.3967	0.5129	6.30E‐15	0.6247	HLA‐DQB1
AA_DQB1_55_32632694_P	32632694	P/A	0.3356	0.4494	6.91E‐15	0.6188	HLA‐DQB1
HLA_DQB1_03	32631061	P/A	0.3356	0.4494	6.91E‐15	0.6188	HLA‐DQB1
AA_DQB1_125_32629935_A	32629935	P/A	0.4646	0.3552	8.81E‐14	1.576	HLA‐DQB1
AA_DQB1_220_32629141_H	32629141	P/A	0.4646	0.3552	8.81E‐14	1.576	HLA‐DQB1
AA_DQB1_220_32629141_R	32629141	P/A	0.4646	0.3552	8.81E‐14	1.576	HLA‐DQB1
AA_DQB1_221_32629138_H	32629138	P/A	0.4646	0.3552	8.81E‐14	1.576	HLA‐DQB1
AA_DQB1_221_32629138_Q	32629138	P/A	0.4646	0.3552	8.81E‐14	1.576	HLA‐DQB1
AA_DQB1_53_32632700	32632700	Q/L	0.4646	0.3552	8.81E‐14	1.576	HLA‐DQB1
AA_DQB1_84_32632607	32632607	E/Q	0.4646	0.3552	8.81E‐14	1.576	HLA‐DQB1
AA_DQB1_85_32632604	32632604	V/L	0.4646	0.3552	8.81E‐14	1.576	HLA‐DQB1
AA_DQB1_86_32632601_E	32632601	P/A	0.4646	0.3552	8.81E‐14	1.576	HLA‐DQB1
AA_DQB1_87_32632598_L	32632598	P/A	0.4646	0.3552	8.81E‐14	1.576	HLA‐DQB1
AA_DQB1_89_32632592	32632592	G/T	0.4646	0.3552	8.81E‐14	1.576	HLA‐DQB1

a Composite and low frequency markers (MAF < 1% in the entire sample set) are not shown.

b Chromosome 6 positions for SNP markers according to genome build hg19.

c P/A: Present/Absent for classical HLA alleles and in the case that a specific that a specific amino acid is given in the Marker ID (see first column).

d Frequency and relative risk (OR) are given for the first allele denoted in the colum “Alleles”.

### 3.3 Independent HLA associations in HLA‐DQB1*0301

We further investigated whether SLE risk was associated with other HLA genes independent of HLA‐DQβ1 amino acid polymorphisms. When conditioning on the most significant amino acid in HLA‐DQβ1 87, we detected significant independent associations at HLA‐DQB1*0301 (*P*_meta_ = 6.91 × 10^−15^, OR = 0.5508) (Figure 1b). After conditioning on HLA‐DQB1*0301 and HLA‐DQβ1 amino acid position 87, no variants in the MHC region satisfied the study‐wide significance threshold (*p* > 1.67 × 10^−6^; Figure 1c; Table S2). These results suggested that polymorphisms in class II HLA genes, particularly HLA‐DQB1, explain the majority of risk for SLE in Chinese Han population.

### 3.4 HLA‐DQB1 amino acid position 87 risks are shared between mainland and Hong Kong

After imputation in data set #2, we found that the effect size and direction of residue at HLA‐DQβ1 amino acid position 87 and HLA‐DQB1*0301 in Hong Kong subjects were highly consistent with the results in mainland (*p* = 5.477 × 10^−4^ and 3.79 × 10^−7^).

## 4 DISCUSSION

Systemic Lupus Erythematosus (SLE) (OMIM 152700) is a polygenic disorder characterized by chronic and systemic inflammation and affecting multiple organs due to a loss of immune tolerance against self‐antigens. The genetic heritability of SLE ranged from 44% to 66% (Lawrence, Martins, & Drake, 1987; Wang et al., 2007). To date, more than 60 susceptibility loci have been identified in genome wide and candidate gene association studies (Boackle, 2013).

Association analysis of HLA genes at amino acid sites have facilitated fine‐mapping efforts in immune‐related diseases (Pereyra et al., 2010; Raychaudhuri et al., 2012). To identify potentially causal variation within HLA genes associated with Chinese SLE patients, we carried out an imputation based on the Han‐MHC reference panel for classical HLA alleles as well as amino acid polymorphisms in mainland and Hong Kong Han SLE patients.

Our results support and refine previous findings of multiple signals in the MHC for population of Asia or European ancestry. By testing HLA alleles, amino acids, and SNPs at the same time, also we were able to pinpoint the amino acid position 11 and 13 of HLA‐DRβ1 as a significant signal in both mainland and Hong Kong Chinese Han populations (*p* = 2.91 × 10^−13^ both), which was recognized as the major risk factor for SLE in Korean (Kim et al., 2014). This may suggest that genetic risk of the MHC region on SLE are generally shared within Asian populations to a certain extent (Kim et al., 2009, 2014). We note that those sites explain part of the variation in MHC‐mediated risk for both populations, and that the residues confer similar directions and relative magnitude of risk. We also observe that allele HLA‐DRB1*15:01 (*p* = 8.08 × 10^−11^) and HLA‐DQA1*0102 (*p* = 2.96 × 10^−10^) in European populations (Morris et al., 2012, 2014) is generally concordant with the results presented here for Chinese Han populations.

HLA‐DRB1 (MIM 142857) alleles are correlated with alleles of the class II loci HLA‐DQA1 (MIM 146880) and HLA‐DQB1 (MIM 604305) as a result of strong LD in the class II region (HLA‐DR and HLA‐DQ). However, amino acid in HLA‐DQB1 have rarely been reported showing association with SLE. In our study amino acid position 87 of HLA‐DQβ1 has the most strongest association with SLE risk, which was firstly been reported till now. Furthermore, HLA‐DQβ1 amino acid position 87 and HLA‐DQB1*0301 in Hong Kong individuals were highly consistent with mainland population. HLA genes encode cell surface proteins that display antigenic peptides to effector immune cells to regulate self‐tolerance and downstream immune responses. Variation in amino acid residues at specific positions within the antigen‐bind grooves may alter the repertoire of presented peptides, and results in the risk of autoimmunity (van Lummel et al., 2014; Scally et al., 2013). A Phe in position 87 (Figure 1d) is located in peptide‐binding grooves of HLA molecules according to our results, suggesting functional contributions to antigen‐ presentation ability or protein stability (Jin et al., 2014; Miyadera, Ohashi, Lernmark, Kitamura, & Tokunaga, 2015). These data suggest the involvement of peptide antigens bound to specific HLA molecules in controlling the development of SLE.

Yusmin et al. (Mohd‐Yusuf, Phipps, Chow, & Yeap, 2011) has ever analyzed HLA alleles in 160 SLE patients (99 Chinese and 61 Malays) and 107 healthy control individuals (58 Chinese and 49 Malays) by sequence specific primer amplification (PCR‐SSP) phototyping techniques, and found that allele frequencies of DQB1*0301 was significantly increased in Malay and Chinese SLE patients compared with healthy control individuals. By performing analyses of HLA‐DQ alleles through conditioning on the HLA‐DRB1 alleles, David et al. (Morris et al., 2012) also found evidence of association independent of DRB1*03:01 and HLA‐DRB1*15:01 (after adjusting for multiple testing) at HLA‐DQB1*03:01 (*p* = 4.61 × 10^−22^) in six studies. Then it can be conclude that HLA‐DQB1*0301 is an important risk factor for SLE patients in Asian population.

In summary, by using imputation as an approach to identify disease risk variants within the MHC region, we determined the population‐specific genetic variants of HLA polymorphisms and also reproduced known HLA alleles and amino acid polymorphisms effects from genotyped SNP data in SLE by using Han‐MHC reference panel. Furthermore, given the prevalence of SLE, our study highlights the role of genetic variants in common diseases.

## CONFLICT OF INTEREST

The authors declare no conflict of interest.
